# Early diagnostic test for acute fatty liver of pregnancy: a retrospective case control study

**DOI:** 10.1186/s12884-020-2787-4

**Published:** 2020-03-14

**Authors:** Yan Zhong, Fufan Zhu, Yiling Ding

**Affiliations:** Department of Obstetrics and Gynecology, The Second Xiangya Hospital, Central South University, No.139 Middle Renmin Road, Changsha, Hunan 410011 People’s Republic of China

**Keywords:** Acute fatty liver of pregnancy, Early diagnosis, Liver function test, Coagulation function test

## Abstract

**Background:**

Early diagnosis is important to lower the mortality rate of acute fatty liver of pregnancy (AFLP). The Swansea criteria is commonly used to diagnose AFLP, but some terms could only be reached when symptoms and signs have progressed, or are not efficient in clinical practice. Therefore, it is necessary to select cost effective tests to simplify and facilitate early suspicion of acute fatty liver of pregnancy.

**Methods:**

This is a retrospective study of 28,800 medical records at the Second Xiangya Hospital from 2009 to 2015, including 41 patients with AFLP and 172 other diseases that could show similar symptoms to AFLP. The evaluated variables included past history of liver diseases, blood pressure, gastrointestinal symptoms, blood count, liver function test, coagulation function test and blood sugar test. The sensitivity, specificity, positive predict value and negative predict value were calculated for models in diagnosing AFLP.

**Results:**

The significant variables associated with AFLP included gastrointestinal symptoms, blood pressure > 140/90 mmHg, aminotransferase> 42 IU/l, total bilirubin> 0.8 mg/dl, total bilirubin acid> 10.0 μmol/L, activated partial prothrombin time(APTT) > 34 s, prothrombin time(PT) > 14 s, white blood cells> 11 *10^6^/l and blood sugar< 72 mg/dl. Gastrointestinal symptoms +aminotransferase +bilirubin +bile acid +APTT/PT showed 97.6% sensitivity and 97.1% specificity to diagnose AFLP. Adding blood pressure, blood sugar or white blood cells decreased the accuracy of the statistical model.

**Conclusions:**

Application of a statistical model including maternal symptoms, biochemical and haematological parameters has high diagnostic accuracy for earlier identification of AFLP. However, this finding needs to be tested in another cohort to determine whether this statistical model has the same performance.

## Background

Acute fatty liver of pregnancy (AFLP) is a rare but potentially lethal condition that typically presents during the third trimester of pregnancy. It is characterized by microvesicular fatty infiltration of the liver and can lead to multiorgan failure [1]. Because of the potential for rapid progression to coma and death, AFLP is considered to be an obstetrics emergency [2–4]. So far, there has been no case of recovery before delivery [5–7]. Reyes [8] reported 100% of AFLP cases could survive if they were delivered within a week after onset of the disease, while 30% would die if they were delivered beyond 2 weeks after onset. Thus, it is important to lower mortality rate by early diagnosis and prompt parturition. However, there is no specific clinical manifestation or diagnostic tests for AFLP, so it is hard to diagnose it early. Once AFLP is diagnosed, serious maternal biochemical or haematological abnormalities might have already resulted, meaning appropriate time for intervention has been lost. Exact diagnosis depends on differentiate diagnosis from diseases that show similar symptoms to AFLP, such as gastroenteritis, severe viral hepatitis, HELLP (hemolysis, elevated serum level of enzymes, and low platelets) syndrome. Women with AFLP mostly present with gastrointestinal symptoms, such as vomiting, nausea and abdominal pain. These symptoms actually are the main reason that made patient come to see doctor and easy to be misdiagnosed with gastroenteritis, etc. The misdiagnosis would delay treatment and lead to fatal results.

The Swansea criteria is commonly used to diagnose AFLP, including vomiting, abdominal pain, polydipsia/polyuria, encephalopathy, bilirubin > 0.8 mg/dl (14 kmol/l), hypoglycemia < 72 mg/dl (4 mmol/l), uric acid > 5.7 mg/dl (340 kmol/l), leukocytosis > 11 *10^6^/l, ascites or bright liver on sonogram, aspartate aminotransferase and alanine aminotransferase > 42 IU/l, ammonia > 27.5 mg/dl (47 kmol/l),creatinine > 1.7 mg/dl (150 kmol/l),coagulopathy (prothrombin time(PT) > 14 s or activated partial thromboplastin time(APTT) > 34 s), microvesicular steatosis on liver biopsy [9]. Six or more of these terms are required to diagnose AFLP. However, some terms could only be reached when symptoms and signs have progressed, such as encephalopathy, ascites or bright liver on sonogram, which might be too late for treatment. In addition, early diagnosis depends a lot on basic prenatal care, while some of the tests might be too expensive or complicate for it, such as liver biopsy, which could also cause complications in the presence of coagulopathy. Therefore, it is necessary to select cost effective tests for early diagnosis.

## Methods

This is a retrospective study in a university obstetric unit. The reference standard diagnosis of AFLP is the Swansea criteria. With approval from institutional human subject review board, we reviewed 28,800 medical records at the Second Xiangya Hospital from 2009 to 2015 and identified 41 AFLP cases diagnosed by Swansea criteria. The incidence of AFLP in our hospital (0.14%) is higher than reported [3, 4],which might be because that our hospital is of the highest level in our province, so most severe cases were transferred here. One hundred seventy-two other diseases that could show similar symptoms to AFLP were used as “control” here, including 40 gastroenteritis, 11 severe viral hepatitis, 40 HELLP syndrome, 41 intrahepatic cholestasis of pregnancy(ICP), 40 ICP with preeclampsia. Gastroenteritis, severe viral hepatitis, HELLP and ICP were defined using International Classification of Diseases, 10th Revision codes. All of the 11 severe viral hepatitis were caused by Hepatitis B Virus.

The significant variables associated with AFLP were selected by univariate analyses and by their biologic plausibility. The candidate variables for the diagnostic model included past history of liver diseases, blood pressure, gastrointestinal symptoms, blood count, liver function test, coagulation function test and blood sugar test. We also tried to further select markers (aminotransferase, bilirubin, prothrombin time (PT), activated partial prothrombin time (APTT), for example) from these tests to make a more exact diagnostic model. The time of test before final diagnosis by Swansea criteria was 3 days. The past history of liver disease referred to chronic liver disease that had been diagnosed but hadn’t been cured before this pregnancy, such as viral hepatitis, autoimmune hepatitis, Wilson’s disease, excluding liver disease that had been cured before this pregnancy, such as acute viral liver disease in childhood or ICP in previous pregnancy. Risk factors that hadn’t resulted in a determined liver disease were not included in the past history of liver disease either, such as alcohol abuse.

Statistical analyses were performed with STATA10.0 (StataCorp, College Station, TX, USA). Descriptive statistics included means and range for continuous variables and frequency distributions for categorical variables. Comparisons between categorical variables were tested by the use of the chi-square test. Variables were included in the model if they were statistically significant in the univariate analyses. Comparisons between normally distributed continuous variables and categorical variables were performed using Student t test and analysis of variance.

## Results

The demographic characteristics for study population are shown in Table 1. All of patients in our study population were Chinese. The significant variables associated with AFLP included gastrointestinal symptoms, blood pressure > 140/90 mmHg, AT> 42 IU/l, TB > 0.8 mg/dl, TBA > 10.0 μmol/L, APTT> 34 s, PT > 14 s, white blood cells> 11 *10^6^/l, blood sugar< 72 mg/dl. At the time of our test, none of the AFLP cases met 6 terms of the Swansea criteria, 19.5% of the cases met 5 terms, 75.6% met 4 terms and 4.9% met 3 terms.

**Table 1:** Demographic characteristics for study population

	AFLP	CC	p
	(*n* = 41)	(*n* = 172)	
Age, mean(SD)	27 (0.4)	30 (0.9)	< 0.05
Gravidity, mean(SD)	2 (0.2)	3 (0.1)	> 0.05
Parity, mean(SD)	1 (0.1)	1 (0.05)	> 0.05
Liver disease history, n(%)	1 (2.4)	19 (11.1)	> 0.05
Gastrointestinal symptoms, n(%)	41 (100)	87 (50.6)	< 0.01
Blood pressure > 140/90 mmHg, n(%)	8 (19.5)	72 (41.8)	< 0.05
AT> 42 IU/l, n(%)	41 (100)	106 (61.6)	< 0.01
TB > 0.8 mg/dl , n(%)	41 (100)	37 (23.3)	< 0.01
TBA > 10.0 μmol/L, n(%)	41 (100)	88 (51.2)	< 0.01
APTT> 34 s, n(%)	39 (95.1)	13 (7.6)	< 0.01
PT > 14 s, n(%)	40 (97.6)	7 (4.1)	< 0.01
White blood cells> 11 *10^6^/l, n(%)	39 (95.1)	53 (30.8)	< 0.01
Blood sugar< 72 mg/dl, n(%)	5 (12.2)	3 (2.3)	< 0.05
Abnormal ultrasound manifestation, n(%)	4 (9.76)	12 (7.0)	< 0.05

*AFLP* acute fatty liver of pregnancy, *CC* case control, *AT* aminotransferase, *TBA* total billirubin acid, *PT* prothrombin time, *APTT* activated partial prothrombin time, *CI* confidence interval

In accuracy analysis, gastrointestinal symptoms +aminotransferase+ bilirubin+ bile acid +APTT/PT showed 97.6% sensitivity and 97.1% specificity to diagnose AFLP. Adding blood pressure, blood sugar or white blood cells didn’t increase the sensitivity but decrease it (Table 2).

**Table 2:** Diagnostic models of early diagnosis for AFLP

Diagnostic model	Sensitivity (%) (95%CI)	Specificity(%) (95%CI)	NPV (%) (95%CI)	PPV (%) (95%CI)
Gastrointestinal symptoms + AT +bile acid +APTT/PT	97.6 (87.1–100)	96.5 (92.6–98.7)	99.4 (96.7–100)	87.0 (73.7–95.1)
Gastrointestinal symptoms + AT + bile acid + APTT/PT + bilirubin	97.6 (87.1–100)	97.1 (93.3–99.0)	99.4 (96.7–100)	88.9 (75.9–96.3)
Gastrointestinal symptoms +blood pressure + AT +bile acid + APTT/PT+ bilirubin	78.1 (62.4–89.4)	97.7 (94.1–99.4)	94.9 (90.6–97.6)	88.9 (73.9–96.9)
Gastrointestinal symptoms + AT +bile acid + APTT/PT + bilirubin + blood sugar	12.2 (4.1–26.2)	98.8 (95.8–100)	82.5 (76.6–87.4)	71.4 (29.0–96.3)
Gastrointestinal symptoms + AT +bile acid+ APTT/PT + bilirubin + white blood cells	92.7 (80.1–98.5)	98.3 (95.0–99.6)	98.3 (95.0–99.6)	92.7 (80.1–98.5)
Gastrointestinal symptoms + AT +bile acid+ APTT/PT + bilirubin + white blood cells +blood sugar	12.2 (4.1–26.2)	99.4 (96.8–100)	82.6 (76.7–87.5)	83.3 (35.8–99.6)

*AT* aminotransferase, *PT* prothrombin time, *APTT* activated partial prothrombin time, *CI* confidence interval, *NPV* negative predict value, *PPV* positive predict value

In all of the 41 AFLP cases, 23 were delivered when our test was positive, while 18 weren’t delivered until at least 6 terms of Swansea criteria were met. Maternal mortality was significantly lower and Apgar scores significantly higher in group delivered by our test comparing to group delivered by Swansea criteria (Table 3).

**Table 3:** Maternal mortality and infant Apgar scores in AFLP cases

	Cases delivered by our test (*n* = 23)	Cases delivered by Swansea criteria (*n* = 18)	p
maternal mortality	0%	33.3%	< 0.05
1 min Apgar scores	6.8 ± 0.6	4.9 ± 0.8	< 0.05
5 min Apgar scores	8.5 ± 0.6	6.8 ± 0.8	< 0.05

## Discussion

Our study showed proper choice of test in basic prenatal care was able to diagnose AFLP. Combination of gastrointestinal symptoms +aminotransferase +bile acid + APTT/PT+ bilirubin showed 97.6% sensitivity and 97.1% specificity, while more markers didn’t necessarily contribute to differentiate diagnosis.

The initial symptoms of AFLP are non-specific gastrointestinal symptoms that overlap symptoms of other diseases in the same trimester, such as gastroenteritis, severe viral hepatitis, HELLP syndrome [3, 10, 11]. Emergent delivery is the first treatment for AFLP, while not necessarily for these diseases [7]. Therefore, it is important to differentiate these diseases from AFLP before treatment, and we chose these diseases as controls. The differential diagnosis mostly based on clinical symptom and laboratory findings. HELLP syndrome is characterized by low platelets, hemolysis and elevated liver enzymes, while low glucose concentration and coagulopathy are less common. ICP is characterized by elevated fasting serum bile acid level, while liver dysfunction is usually mild, and coagulopathy rarely happens. Severe viral hepatitis could show exactly the same clinical presentation as AFLP, including encephalopathy, coagulopathy. Sometimes history of viral hepatitis and viral serum serology test are the only way to differentiate. Gastroenteritis only slightly elevates liver enzymes, without affection on coagulation function. Thus we chose past history of liver diseases, blood pressure, gastrointestinal symptoms, blood count, liver function, coagulant function and blood sugar as markers for differentiation.

Swansea criteria is justified for the presumptive diagnosis of AFLP by high negative predictive value (100%) in diagnosing microvesicular steatosis, but it is not targeted at early diagnosis. On one hand, some of its diagnostic terms could only be reached when symptoms and signs have progressed, which might be too late for treatment. On the other hand, some of its terms are not efficient to apply in clinical practice. For example, although liver biopsy is the gold standard for diagnosis of AFLP, it is likely to cause intraperitoneal hemorrhage in the event of coagulopathy. So seldom has the diagnosis of AFLP been confirmed by liver biopsy in previous reports [10, 12–14]. In our study, none of AFLP cases received liver biopsy because of limited time and risk of hemorrhage. In consistent with previous reports [15–20], our study showed ultrasound was not a reliable method for the diagnosis of AFLP.

To simplify and facilitate early suspicion of AFLP, Goel et al. designed presence of the following three criteria for the presumptive diagnosis of AFLP: 1. late pregnancy (late 2nd or 3rd trimester), 2. Acute liver failure: jaundice with coagulopathy and/or hypoglycaemia and/or encephalopathy, 3. No other explanation for liver failure [7]. The third term indicated the importance of differentiate diagnosis, but it didn’t make in details. Zhu et al. [6] showed the 34th gestation week might be important time for screening AFLP outpatients. Gastrointestinal symptoms, blood routine, liver function, and coagulant function tests were recommended as the first grade screening indicators. Renal function, blood sugar test, and abdominal ultrasound could be the second grade screening indicators for AFLP outpatients. However, they only showed the incidence of the indicators in AFLP cases instead of diagnosis accuracy. Our study also showed gastrointestinal symptoms, liver function test and coagulant function test made the best diagnostic model. We further demonstrated the diagnostic accuracy of the model, and found more markers didn’t necessarily contribute to differentiate diagnosis.

With acceptance of the importance of early recognition and diagnosis of AFLP, maternal mortality has decreased from 85% [21] to 12.5% [5, 12, 16, 17, 22, 23]. However, AFLP still causes severe maternal morbidity and mortality comparing to other pregnancy complications, so earlier diagnosis for outpatient pregnant women could be life-saving [24, 25]. Our study showed there was not enough evidence to diagnose AFLP by Swansea criteria at our time of test, and our test could diagnose AFLP earlier than the Swansea criteria. Maternal mortality was significantly reduced and Apgar scores significantly increased in group delivered by our test comparing to group delivered by Swansea criteria. The result showed early diagnosis by our test could improve both fetal and maternal outcome, although the 3 days ahead doesn’t sound so early. We think it because AFLP is a fatal disease with rapid progression. Similar to emergencies like placenta abruption or acute fetal distress, even a short time we earned could change the prognosis.

**Fig. 1:**
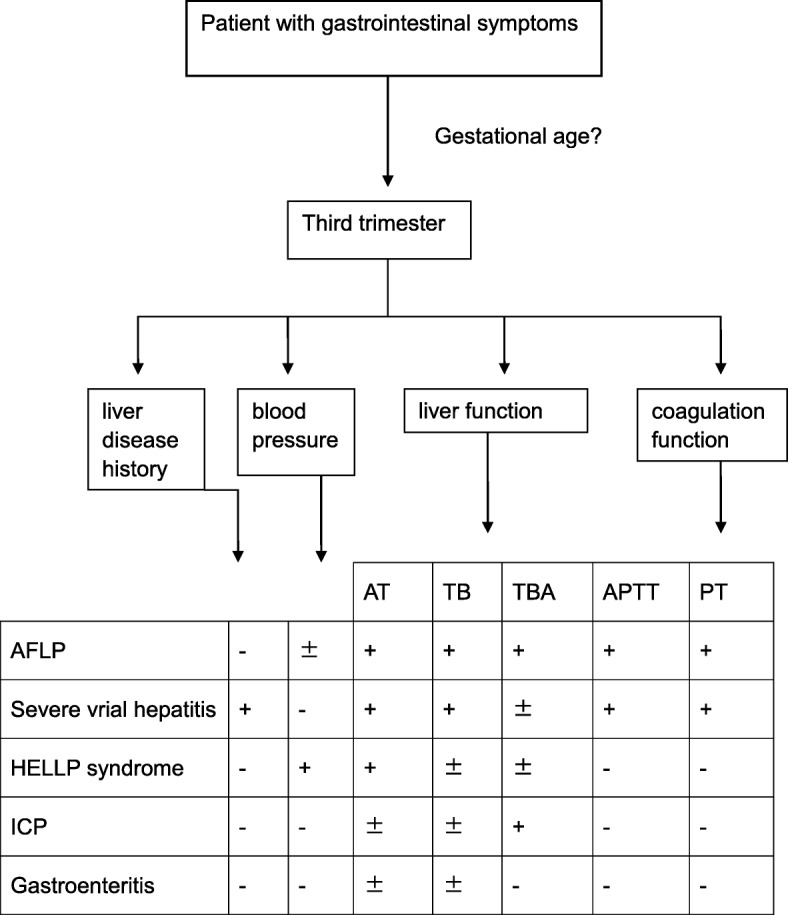
Diagnostic procedure for AFLP. AFLP: Acute fatty liver of pregnancy; HELLP: hemolysis, elevated serum level of enzymes, and low platelets syndrome; ICP: intrahepatic cholestasis of pregnancy; AT: aminotransferase; TBA: total bilirubin acid; PT: prothrombin time, APTT: activated partial prothrombin time, + positive or elevated; − negative or normal

We summarized our design of diagnosis procedure as Fig. 1. For patient in third trimester presented with gastrointestinal symptoms, the possibility of AFLP should always be considered. According to our study, aminotransferase, liver function test and coagulation function test were good ways to differentiate AFLP from other similar diseases. Among patients with gastrointestinal symptoms, if they were found with abnormal liver function and coagulation function test while without liver disease history, the risk of AFLP was high. If they had liver disease history, especially hepatitis B, then AFLP should be diagnosis of exclusion. For liver dysfunction caused by HELLP syndrome, elevated blood pressure should be more obvious and happened earlier, so as elevated bile acid for ICP. Coagulation function test especially APTT and PT was seldom changed for HELLP syndrome or ICP, or would change after hypertension or elevated bile acid was severe enough. Gastroenteritis would affect liver function at the most, without change on coagulation function or blood pressure.

The study is restricted by some limitations. First, the case number is limited and number of every disease in the control group is generally low. Hence, we presented a composite control. Second, the study was carried out in a single hospital. The population was all Chinese who were mostly from central and southern China. So we didn’t have enough data to evaluate the influence of race and geography. Third, the test results were disclosed to the clinicians treating the women, so that clinician’s behavior may have averted diagnoses of AFLP or made them more likely. Thus, we planned to retest the model in double blind study to eliminate the influence of clinician’s behavior. We also plan to evaluate the model in multicenter study and investigate the influence of more confounders.

## Conclusion

Even though AFLP is not common, its diagnosis remains a big challenge for Obstetricians. Our study showed proper choice of test in basic prenatal care was able to diagnose AFLP. Application of a model including maternal symptoms, biochemical and haematological parameters has a high diagnostic accuracy for earlier identification of AFLP. We hope this diagnostic test of AFLP would contribute to simplify and facilitate early suspicion of AFLP. However, the finding needs to be tested in another cohort to determine whether this diagnostic model has the same performance.

## Data Availability

The datasets used and/or analysed during the current study available from the corresponding author on reasonable request.

## Abbreviations

AFLP: Acute fatty liver of pregnancy
APTT: Activated partial prothrombin time
AT: Aminotransferase
HELLP: Hemolysis, elevated serum level of enzymes, and low platelets syndrome
ICP: Intrahepatic cholestasis of pregnancy
PT: Prothrombin time
TB: Total bilirubin
TBA: Total bilirubin acid
